# Supplementary material for: Molecular Modification Enhances Xylose Uptake by the Sugar Transporter KM_SUT5 of Kluyveromyces marxianus
Source: Int J Mol Sci. 2024 Jul 30;25(15):8322. doi: 10.3390/ijms25158322 (PMC11312716; doi:10.3390/ijms25158322)
Supplement: Supplementary file 1 [file ijms-25-08322-s001.zip › ijms-3100334-supplementary.pdf]

Supplementary materials

Molecular modification enhances xylose uptake by the sugar transporter KM_SUT5 of *Kluyveromyces marxianus*

Xiuyuan Luo, Xi Tao, Guangyao Ran, Yuanzhen Deng, Huanyuan Wang, Liyan Tan, Zongwen Pang*

College of Life Science and Technology, Guangxi University, Nanning 530004, China

* **Corresponding author**: Zongwen Pang (E-mail address: pangzw@gxu.edu.cn).

Tel.: +86 771 323 2394

Postal address: College of Life Science and Technology, Guangxi University, Nanning 530004, China

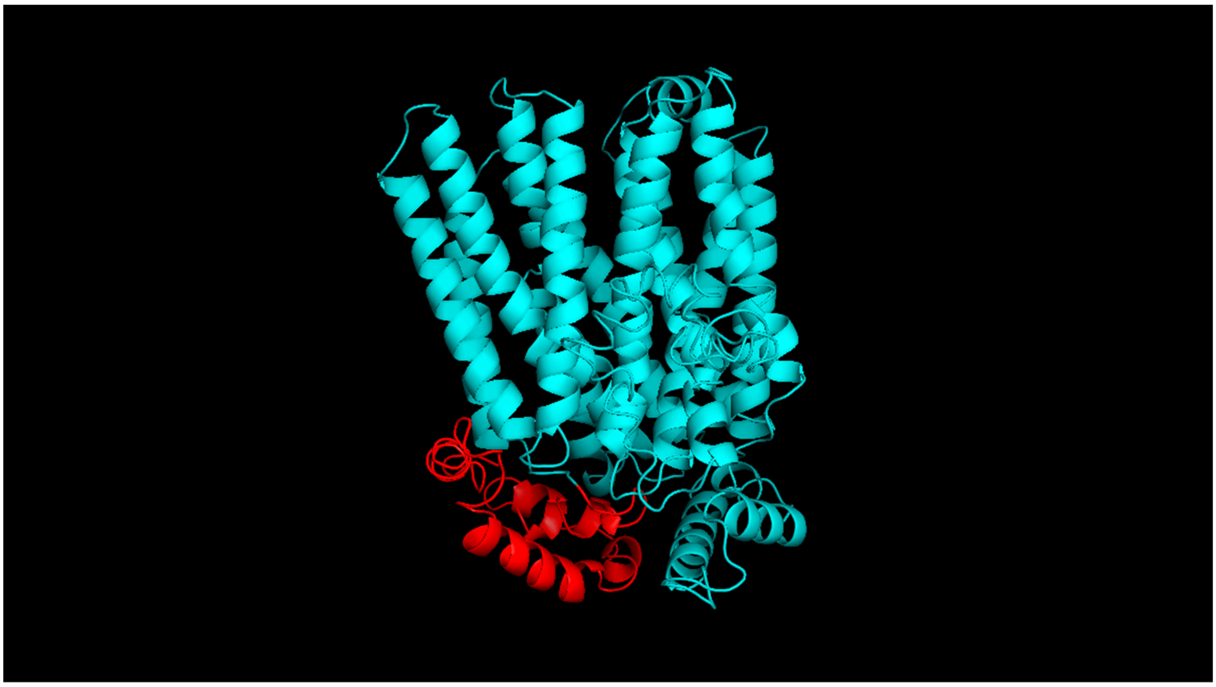

Figure S1: The structure of KM_SUT5p, construct through I-TASSER, analyzed through PyMOL. Red region indicates the C-terminal region of the protein.

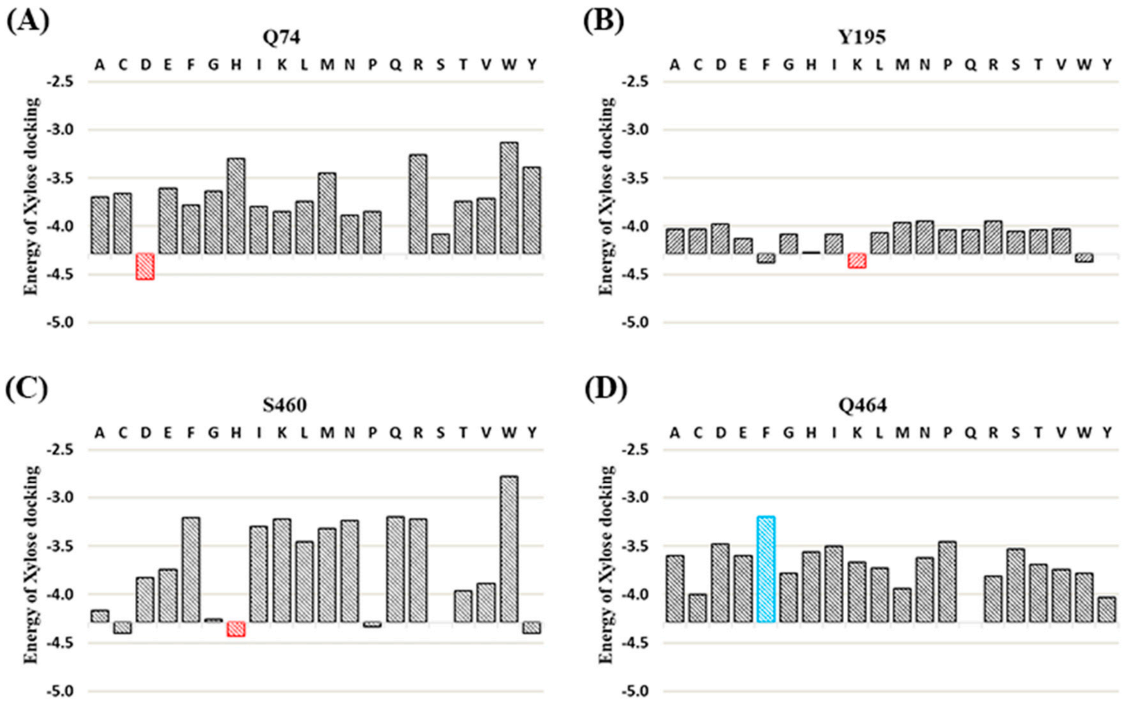

Figure S2: The docking energy of molecular mutation simulation. The red cubic column in (A), (B) and (C) indicates the mutation with the lowest binding energy at this site ,and the blue cubic column in (D) indicates the mutation with the highest binding energy at this site.

Table S1. Strains and plasmids used in this study.

Strain and plasmids names	Description	Source or reference
Strain		
S. cerevisiae EBY.VW4000	MATα leu2-3,112 ura3-52 trp1-289 his3-Δ1 Mal2-8c SUC2 hxt17Δ hxt13Δ::loxP hxt15Δ::loxP hxt16Δ::loxP hxt14Δ::loxP hxt12Δ::loxP hxt9Δ::loxP hxt11Δ::loxP hxt10Δ::loxP hxt8Δ::loxP hxt514::loxP hxt2Δ::loxP hxt367Δ::loxP gal2Δ stl1Δ::loxP agt1Δ::loxP ydl247wΔ::loxP yjr160cΔ::loxP	(Wieczorke et al. 1999)
S. cerevisiae EBY-XYL	*S. cerevisiae* EBY.VW4000, YEplac195-XYL1-XYL2	This study
K. marxianus GX-UN120	mutant strains	(Pang et al., 2010)
S. cerevisiae EBY-P	*S. cerevisiae* EBY-XYL, pRS424-Hxt7	This study
S. cerevisiae EBY-SUT5	*S. cerevisiae* EBY-XYL, pRS424-Hxt7-SUT5	This study
S. cerevisiae Q74D	*S. cerevisiae* EBY-XYL, pRS424-SUT5-74	This study
S. cerevisiae Y195K	*S. cerevisiae* EBY-XYL, pRS424-SUT5-195	This study
S. cerevisiae S460H	*S. cerevisiae* EBY-XYL, pRS424-SUT5-460	This study
S. cerevisiae Q464F	*S. cerevisiae* EBY-XYL, pRS424-SUT5-464	This study
S. cerevisiae delL509-	*S. cerevisiae* EBY-XYL, pRS424-SUT5-509	This study
S. cerevisiae delP519-	*S. cerevisiae* EBY-XYL, pRS424-SUT5-519	This study
S. cerevisiae delV529-	*S. cerevisiae* EBY-XYL, pRS424-SUT5-529	This study
S. cerevisiae delV534-	*S. cerevisiae* EBY-XYL, pRS424-SUT5-534	This study
S. cerevisiae delK544-	*S. cerevisiae* EBY-XYL, pRS424-SUT5-544	This study
S. cerevisiae delA554-	*S. cerevisiae* EBY-XYL, pRS424-SUT5-554	This study
Plasmids		
YEplac195	Shuttle plasmid, URA3, Amp^r	MiaoLingBio,China (P1807)
YEplac195-XYL1-XYL2	Shuttle plasmid, XYL1-XYL2,URA3, Amp^r,	This study
pRS424-Hxt7	Shuttle plasmid, Hxt7p-Hxt7t, Trp, Amp^r	[32]
pRS424-Hxt7-GFP	Shuttle plasmid,Hxt7p-GFP-Hxt7t, Trp, Amp^r	This study
pRS424-Hxt7-SUT5	pRS424-Hxt7, Hxt7p-KM_SUT5-Hxt7t	This study
pRS424-Hxt7-SUT5-GFP	pRS424-Hxt7, Hxt7p-KM_SUT5ΦGFP-Hxt7t	This study
pRS424-SUT5-74	pRS424-Hxt7, Hxt7p-KM_SUT5(Q74D)-Hxt7t	This study

pRS424-SUT5-195	pRS424-Hxt7, Hxt7p-KM_SUT5(Y195K)-Hxt7t	This study
pRS424-SUT5-460	pRS424-Hxt7, Hxt7p-KM_SUT5(S460H)-Hxt7t	This study
pRS424-SUT5-464	pRS424-Hxt7, Hxt7p-KM_SUT5(Q464F)-Hxt7t	This study
pRS424-SUT5-509	pRS424-Hxt7, Hxt7p-SUT5(delL509-taa)-Hxt7t	This study
pRS424-SUT5-519	pRS424-Hxt7, Hxt7p-SUT5(delP519-taa)-Hxt7t	This study
pRS424-SUT5-529	pRS424-Hxt7, Hxt7p-SUT5(delV529-taa)-Hxt7t	This study
pRS424-SUT5-534	pRS424-Hxt7, Hxt7p-SUT5(delV534-taa)-Hxt7t	This study
pRS424-SUT5-544	pRS424-Hxt7, Hxt7p-SUT5(delK544-taa)-Hxt7t	This study
PRS424-SUT5-554	pRS424-Hxt7, Hxt7p-SUT5(delA554-taa)-Hxt7t	This study

Table S2. Primers used in this study

Primer names	Sequence(5`→3`)
SUT5-BamH-F	CGCGGATCCATGGCAGACACT
SUT5-Sma I-R	TCCCCCGGGTCAAACGTACTCC
M13-F	GTAAAACGACGGCCAGT
M13-R	GTCATAGCTGTTTCCTG
SUT5-GFP-F	CGCGGATCCATGGCAGACACT
SUT5-GFP-R	TCCCCCGGGAACGTACTCC
Q74D-F	CTACCATGGACGGctaCAATGCTTC
Q74D-R	GTAGCCGTCCATGGtagAACAGAGG
Y195K-F	GCCGGCCTTAAgaaTACTCTTTGGC
Y195K-R	GTATTCTTAAGGCCGGCAACTttcCC
S460H-F	GTGCATGGAgtgGCTCAGTTCGTTAATC
S460H-R	GAGCCACTCCATGcacAACGTTATTC
Q464F-F	GTGGCtttCTTCGTTAATCAGTTTGC
Q464F-R	CGAAGaaaGCCACTCCAGACACAAC
delL509-F	GGGAAGACTtaaGAAGAGTTGGCTGC
delL509-R	CTCTTCttaAGTCTTCCCTTTAGTTTCG
delP519-F	GAAGCTtaaAACCCAAGAAAGGCATCG
delP519-R	GGGTTttaAGCTTCAAATACAGCAGCC
delV529-F	CGACTGATCCAtaaTTCGCCGCCGA
delV529-R	GGCGAAttaTGGATCAGTCGATGCC
delV534-F	CGCCGCCGAGtaaAGAAGAAATA
delV534-R	CTttaCTCGGCGGCGAAAACTGGATC
delK544-F	GGTtaaAGACAAATCAGAGCGGCTAGAAG
delK544-R	GATTTGTCTttaACCATTTTCCCTGAG
delA554-F	GAAGTGTAtaaAATAGTAACCTTAATGATC
delA554-R	GGTTACTATTttaTACACTTCTAGCC

The underline indicates the inserted restriction site, and the lowercase letters indicate the replaced bases.
